# Computational and neurocognitive approaches to the political brain: key insights and future avenues for political neuroscience

**DOI:** 10.1098/rstb.2020.0130

**Published:** 2021-04-12

**Authors:** Leor Zmigrod, Manos Tsakiris

**Affiliations:** ^1^ Department of Psychology, University of Cambridge, Cambridge, UK; ^2^ Behavioural and Clinical Neuroscience Institute, University of Cambridge, Cambridge, UK; ^3^ Churchill College, University of Cambridge, Cambridge, UK; ^4^ The Warburg Institute, School of Advanced Study, London, UK; ^5^ Department of Psychology, Royal Holloway, University of London, London, UK; ^6^ Department of Behavioural and Cognitive Sciences, Faculty of Humanities, Education and Social Sciences, University of Luxembourg, Luxembourg

**Keywords:** political cognition, political neuroscience, computational cognitive science, ideology, social neuroscience, political psychology

## Abstract

Although the study of political behaviour has been traditionally restricted to the social sciences, new advances in political neuroscience and computational cognitive science highlight that the biological sciences can offer crucial insights into the roots of ideological thought and action. Echoing the dazzling diversity of human ideologies, this theme issue seeks to reflect the multiplicity of theoretical and methodological approaches to understanding the nature of the political brain. Cutting-edge research along three thematic strands is presented, including (i) computational approaches that zoom in on fine-grained mechanisms underlying political behaviour, (ii) neurocognitive perspectives that harness neuroimaging and psychophysiological techniques to study ideological processes, and (iii) behavioural studies and policy-minded analyses of such understandings across cultures and across ideological domains. Synthesizing these findings together, the issue elucidates core questions regarding the nature of uncertainty in political cognition, the mechanisms of social influence and the cognitive structure of ideological beliefs. This offers key directions for future biologically grounded research as well as a guiding map for citizens, psychologists and policymakers traversing the uneven landscape of modern polarization, misinformation, intolerance and dogmatism.

This article is part of the theme issue ‘The political brain: neurocognitive and computational mechanisms'.

## 1. Unravelling the roots of ideological behaviour

The inherent challenge—and exciting promise—of political psychology and neuroscience is the task of investigating an endlessly intricate organ (the brain) in wildly diverse social contexts (the arena of ideologies). These complexities naturally compound each other, rendering a robust psychological science of ideologies and political behaviour both challenging and crucial. The rapid spread of misinformation propagated by digital media as well as pronounced tribalistic polarization within and between national entities has provoked a global sense that our understanding of the origins of voting behaviour and ideological worldviews is dangerously insufficient. While the study of political attitudes and behaviour has been traditionally confined to the social sciences, new advances in political neuroscience and computational cognitive science highlight that the biological sciences may offer crucial insights about political and ideological behaviour. Ideological behaviour can be defined as behaviour that is epistemically dogmatic and interpersonally intolerant towards non-adherents or non-members [1]. In other words, a person thinking or behaving ‘ideologically' is rigidly adhering to a doctrine, resisting credible evidence when forming opinions, and selectively antagonistic to individuals who do not follow their ideological group or cause. Ideological behaviour can therefore occur in the realm of politics, religion, gender, race, class, social media or any other area of life where social conditions are described and accordingly actions are narrowly prescribed, resulting in ingroups and outgroups.

Yet what prompts an individual to behave ideologically? What neurocognitive processes are underway when a person evaluates socio-political information and comes to dogmatic conclusions? Why do some people fall into the traps of polarization more easily than others? These are some of the pertinent questions that a science of the political brain aims to elucidate and critically evaluate.

Until recently, social psychology was fairly limited in the methods available to study such processes rigorously. Measurement approaches were overwhelmingly based on self-report questionnaires, which are susceptible to self-knowledge and social desirability biases and which struggle to tap into unconscious processes and dispositions. Methods were restricted to laboratory-based studies, encompassing participants from limited university student populations that were frequently neither representative nor diverse. Nonetheless, over the last decade, and the past 5 years in particular, the methodology has advanced beyond recognition. Behavioural and cognitive measures can now be administered online, allowing for genuine interdisciplinarity between political research questions and cognitive methods that quantify implicit psychological processes and traits. These effective online paradigms possess the added value of increasing the accessibility and diversity of participant populations and enabling more reliable cross-cultural comparisons. Moreover, the advent of neuroscience has opened up the field towards studying the neural systems that underpin political cognition, resulting in both new insights and dangerous pitfalls [2,3]. Computational modelling approaches are also now sliding into political psychology [4], facilitating more precise calculation of cognitive parameters as well as more imaginative and complex simulations of how mental processes interact with social dynamics. Political psychology and neuroscience have therefore never been better placed to address the nuances of the political brain. At a time of substantial ideological turmoil and division, the field has also perhaps never been more pertinent.

Yet—as any good philosopher of science will observe—improved methodologies have a limited impact without inventive and thoughtful theoretical approaches that can take the field forward and build knowledge across disciplines. It is this marriage between cutting-edge methods and original, well-reasoned hypotheses that this special issue wishes to highlight. This collection of state-of-the-art research in political neuroscience, psychology and political science seeks to illustrate that a robust science of political behaviour is possible and productive, illuminating critical insights about the nature of ideology, the human brain and the societies we live in.

We have chosen to highlight three strands of research: (i) computational approaches that zoom in on fine-grained mechanisms underlying political behaviour, (ii) neurocognitive perspectives that harness neuroimaging and psychophysiological techniques to study ideological processes, and (iii) behavioural studies and policy repercussions of such understandings across cultures and across ideological domains. Evaluating these interdisciplinary approaches together unearths common themes that can inform present theory as well as guide future research efforts. These will be synthesized and summarized below. Above all, we hope that the empirical findings and theoretical implications presented in this theme issue will inspire researchers, policy makers and scholars from a range of disciplines to tackle the intricacies of studying brains in their political environments with rigour, innovation and hope.

## 2. Computational approaches

Computational perspectives on the nature of ideological behaviour typically take two primary forms: computational simulations of hypothetical behavioural dynamics or computational modelling of human behaviour on cognitive tasks. The papers in this collection reflect both types of computational approaches and result in striking overlaps and complementary findings.

Kashima *et al*. [5] explore how computational models of social influence in networks relate to ideological discourse. Using a computational model of communication, the authors identify four subtypes of potential ideological agents according to their level of cognitive bias and motivational ego-involvement when interpreting and storing information in memory. This maps on to the doctrinal and relational components of ideological thinking posited by Zmigrod [1]. The results demonstrate that certain kinds of ideological minds are more likely to polarize in particular ways and that even non-ideological agents can polarize if they communicate exclusively with polarized agents. Hence, the computational modelling employed by Kashima *et al*. [5] illustrates the subtle ways in which cognitive dispositions can interact with political contexts to shape the course of polarization. As they conclude, micro-psychological and macro-historical processes modulate each other in profound ways.

In another demonstration of the interaction between psychological mechanisms and interpersonal dynamics, De Dreu *et al*. [6] review the literature on how agents in political conflict can be modelled through a game theory framework informed by neurobiological insights. Synthesizing formal models with the literature on the neurocognitive roots of attack and defence strategies, the authors argue that the likelihood of status quo revision can be predicted by understanding a host of psychological processes, including the nature of selfish and non-selfish motivations, information-processing capacity to compute cost-benefit trade-offs and metacognitive beliefs.

Metacognition is dissected further by Rollwage & Fleming [7], who use simulation-based modelling to demonstrate that metacognitive insight modulates the adaptiveness of confirmation bias. Agents with accurate metacognitive skills can in fact *benefit* from biased information processing, suggesting that confirmation bias itself may only be deleterious for individuals who also have a metacognitive impairment. Metacognitive ability may thus be a useful locus for interventions aiming to reduce dogmatism and belief polarization.

To elucidate the cognitive basis of dogmatic and ideological thinking, Zmigrod *et al*. [8] conducted a large-scale data-driven investigation. By administering 37 cognitive tasks and 22 personality surveys, and studying the links to 16 ideological attitudes, Zmigrod and colleagues [8] examined how psychological dispositions sculpt individuals' ideological worldviews. Through computational drift-diffusion and Bayesian modelling, the researchers found that individuals' ideologies mirrored their cognitive decision-making strategies. Dogmatism was characterized by impaired evidence accumulation in perceptual decision-making tasks as well as impulsive personality, revealing that dogmatism may emerge owing to general tendencies to make impulsive decisions based on imperfectly processed evidence. Furthermore, the findings illuminate the cognitive and personality roots of political conservatism, nationalism, authoritarianism, system justification, social dominance orientation and extremist attitudes. It is therefore a key resource for scientists of ideology interested in the psychological individual differences that give rise to ideological thought and action.

The underpinnings of political behaviour are further explicated by Lau's [9] review of the literature on social categorization as latent structure learning. The paper argues that in order to understand political phenomena, scientists must adopt high-level conceptualizations of social categorization. Lau's research on how the brain probabilistically infers and tracks latent groups demonstrates that individuals' assessments of the contours of their group identities rely not only on how similar they are to the targets, but on a whole host of contextual factors. The manner in which individuals understand their ingroups and outgroups is thus more complex than previously imagined and can shed light on political polarization in various parliamentary structures—as well as its antidotes.

## 3. Neurocognitive systems

How the brain computes membership to multiple groups can also be unpacked through neuroscientific endeavours, as exemplified in the functional magnetic resonance imaging (fMRI) study conducted by Krosch *et al*. [10]. In their research on how the brain processes race categories, they examine how political ideology modulates racial categorization. In a politically diverse sample of white participants, Krosch *et al*. [10] explored *hypodescent*, a type of social discrimination whereby multiracial individuals are categorized in terms of their ‘socially subordinate’ racial group. The researchers found that conservatives' use of hypodescent was mediated by heightened anterior insula activity occurring when categorizing racially ambiguous faces. A neural sensitivity to racial ambiguity—and not necessarily racial animus against black individuals—may therefore be an important process underlying toxic and discriminatory behaviour. This highlights the value of using neuroimaging approaches to deconstruct ideological phenomena.

The critical role of uncertainty in the neural mechanisms underpinning ideological behaviour was innovatively explored by Haas *et al*. [11]. In an fMRI paradigm that presented participants with leaders' policy positions that were either congruent or incongruent with the political candidate's stated party, and which were marked by variable levels of certainty, Haas *et al*. [11] analysed the ways in which political evaluation is modulated by uncertainty and ideological congruence. Similarly to Krosch *et al*.s' [10] findings, the study implicated heightened activation of the insular cortex, as well as the anterior cingulate cortex, in response to policy positions that were certain but incongruent with the political candidate's party affiliation. By contrast, diminished activation in the bilateral insula was evident when the policy statement was certain and ideologically congruent. Consequently, uncertainty and congruency interact to shape neural and behavioural responses to leaders' policy stances, underscoring that the brain's sensitivity to uncertainty modulates its experience of the political world.

In a different—and unique—kind of methodological approach to the neurocognitive systems instigating political behaviour, Nam *et al*. [12] studied patients with frontal lobe lesions, amygdala lesions and healthy controls. The presence and size of frontal lesions were specifically associated with political conservatism, suggesting that frontally mediated processes may be key for liberal ideologies. Interestingly, patients with anterior temporal lobe lesions were as liberal as healthy control participants. This points to the importance of studying both classically frontal executive functions as well as amygdala-mediated emotional processes in order to understand the complex links between biology, cognition and ideology. Rare lesion studies, such as those conducted by Nam *et al*. [12], as well as complementary approaches in healthy populations, may be critical steps forward in this regard.

Moore *et al*. [13] also hypothesized that the prefrontal cortex may be specifically activated in the context of political belief evaluation. In an fMRI paradigm that resembles Haas *et al*.s' [11] approach, British participants were presented with Brexit-related tweets and rated their belief and emotional valence about those tweets. The belief task activated areas associated with self-referential judgements whereas rating one's emotional response to the political information engaged a broader range of neural regions, including frontal and parietal areas, but notably no prefrontal cortex activation. How and why the prefrontal cortex matters for political cognition therefore remains an open question. Moore *et al*. [13] offer an important discussion about the extent to which these neuroimaging results bear on different theoretical accounts of misinformation acceptance.

Relying on psychophysiological approaches to embodiment and brain functions, Tsakiris *et al*. [14] examine the theoretical and empirical basis for a study of politics that puts visceral processes at its heart. Tsakiris *et al*. [14] discuss the intersection of interoceptive inference, emotions and politics, and argue that physiological signals may influence political behaviour. Through a proof-of-concept study, the researchers suggest that inducing physiological arousal can impact individuals' inclinations towards less authoritarian leaders, illuminating the potential mechanisms governing visceral politics. As Tsakiris *et al*. [14] conclude, understanding how biological anxiety translates into ideological behaviour can shed light on both historical events as well as humans' reactions to future existential threats.

The ways in which biology can be maladaptively co-opted for ideological purposes is also investigated by Saguy *et al*. [15] who hone in on gender ideology and its roots in biological essentialism. Taking a more meta-theoretical approach to neurocognitive systems, Saguy *et al*. [15] argue that non-egalitarian gender ideologies prosper when gender differences are conceptualized in a biologically essentialist manner. Assumptions that men and women differ neurally, hormonally and behaviourally have often been used as tools for the oppression of women—both by and against women—and this categorical distinction between the genders feeds into cognitive biases that reinforce such binaries. Saguy *et al*. [15] therefore posit a gender-binary cycle which can only be cut through a psychological understanding of cognitive biases, motivations and identities as well as an overarching aim to achieve gender equality. The researchers' focus on biological essentialism also serves as an important warning for political psychologists dealing with other kinds of ideological identities: we need to ensure that in studying the biological roots of ideology, we do not reinforce false binaries between political groups that later translate into greater polarization.

## 4. Behavioural paradigms and policy implications

In an effort to tackle potential sources of polarization, Lees & Cikara [16] disentangle actual from false polarization, where the latter stems from inaccurate perceptions about how groups perceive each other. Lees & Cikara [16] offer a typology of polarization and outline how various polarization phenomena can be studied at the level of the individual and society, across ideological issues, partisan identification and prejudice. Furthermore, using a componential analysis of group meta-perceptive accuracy, the researchers identify specific deficits in estimating outgroup members' beliefs, even in cases when the overall interpretation of polarization estimates are accurate. Differentiating between ingroup and outgroup beliefs and between first- and second-order beliefs is shown to be theoretically and empirically useful. Lees and Cikara's contribution is thus an essential resource for researchers concerned with specific forms of polarization and their psychological origins.

Ecker *et al*. [17] also tackle the nature of polarization but focus on whether partisan identities shape misinformation processes. In a behavioural study measuring individuals' processing of political misinformation corrections, Ecker *et al*. [17] found that retractions are effective in reducing individuals' reliance on misinformation. Moreover, individuals' worldviews have an effect on their reasoning—people respond more strongly to worldview-congruent relative to worldview-incongruent information. Notably, conservatives and liberals responded equally to misinformation retractions, regardless of whether the misinformation confirmed or challenged their worldview. This contributes to an emerging scientific debate regarding whether liberals are less susceptible to misinformation than conservatives—and what psychological dispositions make individuals resilient in the face of propaganda and ideologically polarizing information.

In a review of the literature on the socio-cognitive processes of radicalization, Belanger [18] posits that viewing ideological obsession as akin to other forms of addictions may be a fruitful parallel. Violent extremism emerges as an addiction to a belief system and is amplified by the loss of personal significance, suppression of alternative goals, and ego-defensiveness. Considering ideological obsession in this way has the potential to clarify the commonalities across a range of ideological issues, from environmentalism to religious fundamentalism, and from social activism to political conservatism. Belanger's [18] approach is also critical for policymakers seeking to decelerate radicalization and offer credible counter-narratives that do not backfire and accidentally lead to greater extremism as a result. The review illustrates that incorporating insights from human learning can allow practitioners to substitute ideological obsession with alternative, healthier goals that provide existential meaning but do not cause interpersonal harm or disorder.

Arceneaux *et al*. [19] examine a related construct to ideological obsession but one that emerges from an obsession with status and disruption: need for chaos. In four separate panel studies in the UK, USA, Canada and Australia, spanning over 12 000 participants, the researchers explored the prevalence and psychological structure of need for chaos. Latent profile analysis revealed that the desire to ‘watch the world burn' has varied manifestations and origins among people, with some motivated by a wish to rebuild society into better forms and others driven by a nihilistic enjoyment of destruction itself. Evaluating how the frustrations of these subgroups can be met and replaced by healthier attitudes may thus be a pertinent political task in order to ensure that democracy is not hijacked by those intending to destroy it.

Investigating the psychology of what brings people to cooperate, Romano *et al*. [20] conducted a large cross-cultural study of 18 000 participants in 42 nations. Using a classic behavioural economics paradigm, the Prisoner's Dilemma game, Romano *et al*. [20] manipulated the nationality of the game partner in order to evaluate the relationship between national parochialism, political ideology and trust. The results reveal that political ideology modulated cooperative behaviour—liberals tended to display more cooperation and trust than conservatives and expected more cooperation from strangers in general. Importantly, this relationship was dependent on cultural context: ideological differences were pronounced in wealthy nations with high levels of government effectiveness and rule of law. Cross-cultural comparisons can thereby reveal both the generalizability and limits of political psychology insights, emphasizing that culture and context matter.

Finally, the theme issue culminates with an opinion piece by Rovira Kaltwasser [21] focused on how to bridge political psychology with the political study of populism. Highlighting the value of interdisciplinarity between political science, policy and psychology, Rovira Kaltwasser [21] discusses how the psychology of political identities and conspiracy theories can inform—and be informed by—scholarship on populism. The biological sciences and the political sciences can therefore be harmonized in order to illuminate the roots of us-versus-them mentality, system justification and voter mobilization. The contributions of this theme issue both exemplify these interdisciplinary insights and push for new frontiers and methodological possibilities.

## 5. Looking forward: political brains and brainy politics

Echoing the dazzling diversity of ideologies that exist in human societies, this theme issue sought to reflect the multiplicity of theoretical and methodological approaches to understanding the nature of the political brain. Needless to say, investigating such complex processes is fraught with challenges, and it is for this reason that we aimed to exhibit a selection of the most innovative and cutting-edge research being conducted in the field. Computational approaches offer the promise of precision, neurocognitive perspectives hold the potential to add biological and mechanistic depth, and behavioural studies reveal how these dynamics play out in varied social contexts and can be used for constructive policy and political cooperation.

Several recurring themes caught our eye when synthesizing these multifaceted approaches. Unpacking the nature of *uncertainty* and how it contributes to the brain's processing of race [10], political policies [11] and misinformation [13] is a fertile ground for the future of political neuroscience. Indeed, understanding how the brain deals with ambiguous external and internal signals about group membership [9], group meta-perceptions [16] and political emotions [14] could hold clues about how and why citizens are swayed towards parochialism, polarization and authoritarianism.

Linked to this, several lines of research converged on the mechanisms of *social influence*, positing how it affects individuals differentially based on their cognitive traits [5–7], motivations [19] and cultural context [20]. Toxic ideologies become internalized by individuals through cyclical [15] and addictively self-reinforcing [18] mechanisms, and it is these socio-cognitive loops that practitioners and equality advocates may need to break in order to achieve ideological open-mindedness and receptivity to evidence. Social influence can thus be a force for good—regardless of baseline ideological inclinations [17]—when it is used to create balanced information ecosystems that are resilient to misinformation and populism [21].

Lastly, these approaches can shed light on the *cognitive structure of ideological beliefs*, illustrating that there may be core neural, perceptual and cognitive dispositions that facilitate ideological dogmatism, extremism, or conservatism [5,8,10,12,14,18]. Computational and neurocognitive methodologies can thus unearth the psychological processes that govern adoption of intolerant ideological worldviews, even when these are implicit or invisible to the naked eye. Consequently, anatomizing ideology through the lens of psychology and neuroscience can bring to light underlying structures and processes that a macro-historical—or even a purely behavioural approach—might obscure or hide.

The research presented here not only illuminates the political brain and how it functions when it is bombarded by the ambiguities and contradictions of ideologies—it also hints at what an informed, evidence-based (brainy) approach to politics could look like. Understanding the tensions between cognitive biases, emotional heuristics, perceptual corridors and socio-political contexts may be essential for politicians, policymakers, and the public as they navigate the bumpy terrain of modern democracies and tyrannies. Political psychology and neuroscience therefore can and should serve both science and societies in the fight against intolerance, dogma and propaganda.

## Data accessibility

This article has no additional data.
